# Diabetes and prediabetes among women universally screened for gestational diabetes: a multi-ethnic, population-based, prospective study with eleven years follow-up

**DOI:** 10.1186/s12889-025-22493-x

**Published:** 2025-04-03

**Authors:** Christin W. Waage, Anne Karen Jenum, Ibrahimu Mdala, Sindre Lee-Ødegård, Anja Maria Braend, Line Sletner, Jens Petter Berg, Kåre I. Birkeland

**Affiliations:** 1 Department of General Practice, Institute of Health and Society, General Practice Research Unit (AFE), University of Oslo, Post Box 1130 Blindern, Oslo, N- 0318 Norway; 2 Department of Rehabilitation Science and Health Technology, Faculty of Health Sciences, Oslo Metropolitan University, Oslo, Norway; 3 Institute of Clinical Medicine, Faculty of Medicine, University of Oslo, Oslo, Norway; 4 Department of Endocrinology, Obesity and Preventive Medicine, Oslo University Hospital, Oslo, Norway; 5 Department of Medical Biochemistry, Oslo University Hospital, Oslo, Norway

**Keywords:** Gestational diabetes, Type 2 diabetes, Ethnicity

## Abstract

**Background:**

Gestational diabetes (GDM) is a strong risk factor for later development of diabetes. However, data are scarce on the long-term risk for diabetes or prediabetes diagnosed by HbA1c, in non-selected, multi-ethnic populations universally screened for GDM using the WHO_2013_ criteria. We aimed to investigate the development of diabetes or prediabetes eleven years after the index pregnancy and identify risk factors in pregnancy or shortly after.

**Methods:**

A population-based cohort study of 360 women with complete eleven years follow-up data for diabetes (HbA1c ≥ 48 mmol/mol) or prediabetes_ADA_ (HbA1c 39–47 mmol/mol). Women were enrolled in gestational week 15 and universally screened with an oral glucose tolerance test in week 28. We performed least absolute shrinkage and selection operator (LASSO) regression to identify predictors of future diabetes or prediabetes_ADA_ and constructed a nomogram to predict individual risks.

**Results:**

Diabetes or prediabetes_ADA_ combined, was found in 26.9%, and the prevalence was slightly higher in previous GDM compared with non-GDM women (35.6% versus 23.5%; *p* = 0.019). The relative risk (RR) for developing diabetes or prediabetes_ADA_ was moderately elevated in GDM compared with non-GDM women (1.4 [1.0, 1.9], *p* = 0.035). Seven women (1.9%) had diabetes and all of these except for one, had previous GDM. Hence, the crude prevalence was 5.8% among GDM women vs. 0.4% among non-GDM women. The RR for developing diabetes was substantially higher in GDM vs. non-GDM women (14.8 [2.6, 277.1], *p* = 0.012). Prediabetes_ADA_ was found in 25% and the RR for prediabetes_ADA_ was not significantly increased for GDM compared to non-GDM women (1.3 [0.9, 1.8], *p* = 0.143). Among Europeans, 17.0% had diabetes or prediabetes_ADA_, compared to 43.0% among South Asians (*p* < 0.001) and 34.4% among other ethnicities (*p* = 0.002). The most significant predictors identified from the LASSO were HbA1c measured in early pregnancy, ethnicity, and a family history of diabetes.

**Conclusions:**

The risk for developing diabetes was low, overall and among GDM women. Still GDM represented a strong risk for diabetes, but not for prediabetes_ADA_. HbA1c early in pregnancy, non-European ethnicity, and a family history of diabetes were the strongest risk factors for developing diabetes or prediabetes_ADA_.

**Trial registration:**

STORK G2 Women and Risk of Diabetes. NCT03870724 (ClinicalTrials.gov). February 27th, 2019.

**Supplementary Information:**

The online version contains supplementary material available at 10.1186/s12889-025-22493-x.

## Background

Gestational diabetes mellitus (GDM) and diabetes share genetic and environmental risk factors [1, 2]. GDM is considered as an early marker of disturbances in the glucose metabolism [3], and women diagnosed with GDM have increased risk of developing diabetes later in life [4]. However, the reported relative and absolute risks for developing diabetes after GDM vary widely, due to differences in diagnostic criteria and recommended screening procedures [5]. Populations examined also vary largely in ethnic and genetic background, exposure to unhealthy lifestyle and length of follow-up [6, 7]. Few studies have reported diabetes and prediabetes evaluated by HbA1c only, the currently most used diagnostic test.

The original criteria for screening and diagnosis of GDM primarily focused on identifying women at high risk for development of diabetes after pregnancy [8, 9]. However, the current criteria aim to prevent complications in mother and child in the actual pregnancy [10, 11]. The WHO_2013_ diagnostic criteria for GDM have a lower threshold for fasting plasma glucose (FPG) compared to the previous WHO_1999_ criteria. Hence, more women are diagnosed with GDM and are in the need for follow-up during and after pregnancy. Moreover, several stakeholders, including the American Diabetes Association (ADA), now recommend universal screening for GDM with an oral glucose tolerance test (OGTT) in gestational week 24–28 [12], in contrast to high-risk screening as previously used. The adverse effects of slightly elevated glucose in pregnancy for the mother and the newborn has been thoroughly described [13]. However, there is far less data on the long-term risk for diabetes or prediabetes in the mother after universal screening for GDM using the WHO_2013_ criteria.

We have previously reported a high prevalence of GDM by the WHO_1999_ criteria, and substantially higher by the WHO_2013_ criteria, in the STORK Groruddalen (STORK G) cohort, set up to explore short and long-term ethnic differences in maternal health, primarily GDM, and effects of exposures on risk for type 2 diabetes, cardiovascular disease and other health issues [14]. In this population-based, multi-ethnic cohort of pregnant women, we aimed to investigate the development of HbA1c-diagnosed diabetes or prediabetes eleven years after the index pregnancy in which they underwent universal screening for GDM. We also aimed to identify risk factors that predicted later diabetes or prediabetes, as this information may be used to identify women who would benefit from targeted follow-up.

## Methods

The primary outcome was diabetes (HbA1c ≥ 48 mmol/mol (≥ 6.5%)) or prediabetes_ADA_ (HbA1c 39–47 mmol/mol (5.7–6.4%)) [15] eleven years after the index pregnancy, stratified by GDM status and ethnic group.

### Baseline examinations 2008–2011

The population-based STORK G cohort was set up in 2008–2010 at three primary care Child Health Clinics in Groruddalen, Oslo, Norway, a district with a high proportion of ethnic minorities mostly from Asia and Africa [16]. To facilitate inclusion of ethnic minority women often excluded from research, information material and questionnaires were provided in eight languages: Arabic, English, Sorani, Somali, Tamile, Turkish, Urdu, and Vietnamese. Healthy pregnant women attending antenatal care were invited to participate, if they (1) were living in one of the districts, (2) planned to give birth at the two nearby study hospitals (Akershus University Hospital and Oslo University Hospital, Ullevål), (3) were in gestational week < 20, (4) were not suffering from diseases necessitating intensive hospital follow-up during pregnancy, (5) were not already included with a pregnancy lasting > 22 weeks, (6) could communicate in Norwegian or any of the other eight languages, and (7) were able to give informed consent [16]. A rich dataset including questionnaire data, physical measurements and fasting venous samples from gestational week 15 (visit 1), gestational week 28 (visit 2), and 14 weeks postpartum (visit 3) was collected by trained study personnel. Of the 823 healthy pregnant women included (74% of invited), 59% had ethnic minority background, and the sample was found to be representative of those invited [16, 17].

Fasting blood samples were collected at all visits [1–3] after an overnight fast, analysed consecutively at Akershus University Hospital and the Hormone Laboratory, Oslo University Hospital, or were biobanked and stored at -80 °C. FPG values were measured from venous blood on gel tubes, allowed to clot for 30 min before cells were separated from serum, stored at + 4 °C and daily shipped and handled at the Department of Multidisciplinary Laboratory Medicine and Medical Biochemistry, Akershus University Hospital (Vitros 5.1 FS, Ortho Clinical Diagnostics, slide adapted colorimetric method). HbA1c was measured in venous EDTA blood with HPLC (Tosoh G8, Tosoh Corporation) [18]. Fasting total triglycerides were analysed in serum with a colorimetric method (Vitros 5.1 FS, Ortho clinical diagnostics). Leptin and C-peptide were measured at the Hormone Laboratory, Oslo University Hospital, leptin with radioimmunoassay from Millipore Corp (formerly from Linco Research Inc) and C-peptide by non-competitive immunofluorometric assays (DELFIA, PerkinElmer Life Sciences, Wallac Oy, Turku, Finland). Insulin resistance (HOMA-IR) and β-cell function (HOMA-β) were estimated by the Oxford University HOMA Calculator 2.2 from FPG and C-peptide concentrations [19].

In gestational week 28, all women were given a standard 75 g oral glucose tolerance test (OGTT) after an overnight fast [17]. Fasting at 2-h glucose was measured on site in venous EDTA blood, using a patient-near method (HemoCue 201+, Angelholm, Sweden). In total, 99 women were diagnosed with GDM by WHO_1999_ criteria of whom only five needed pharmacological glucose lowering treatment during pregnancy, and none had diabetes at the 14-week postpartum visit. In this study, however, we primarily use the new WHO_2013_ criteria to define GDM (FPG ≥ 5.1 mmol/l or 2-hour glucose ≥ 8.5 mmol/l), hereafter called GDM_WHO2013_, but also report results for the WHO_1999_ criteria (FPG ≥ 7.0 and/or 2-hour glucose ≥ 7.8 mmol/l), and hereafter called GDM_WHO1999_.

Maternal age at inclusion was calculated based on date of birth. Parity was categorised as nulliparous or multiparous (≥ 1), referring to status before the index pregnancy. Education was categorised as lower level (< 12 years) or higher level (≥ 12 years) [14]. Family history of diabetes was self-reported and categorized as yes or no. Height was measured to the nearest 0.1 cm using a fixed stadiometer at inclusion. Body weight was measured with a bioelectrical impedance analysis scale (Tanita-BC 418 MA, Tanita Corporation, Tokyo, Japan) [20], and body mass index (BMI) (kg/m^2^) was calculated.

Ethnicity was defined based on self-reported country of origin of the woman or her mother, if the participant’s mother was born outside Europe or North America [14]. We thereafter merged the largest groups and used the following categories: European, South Asian (primarily from Pakistan and Sri Lanka), and other ethnicities (mostly from Middle Eastern countries, Eastern Asia and Africa). Only 6.4% of non-Europeans (*n* = 23) were second-generation immigrants.

### Follow-up examinations 2019–2022

**Fig. 1:**
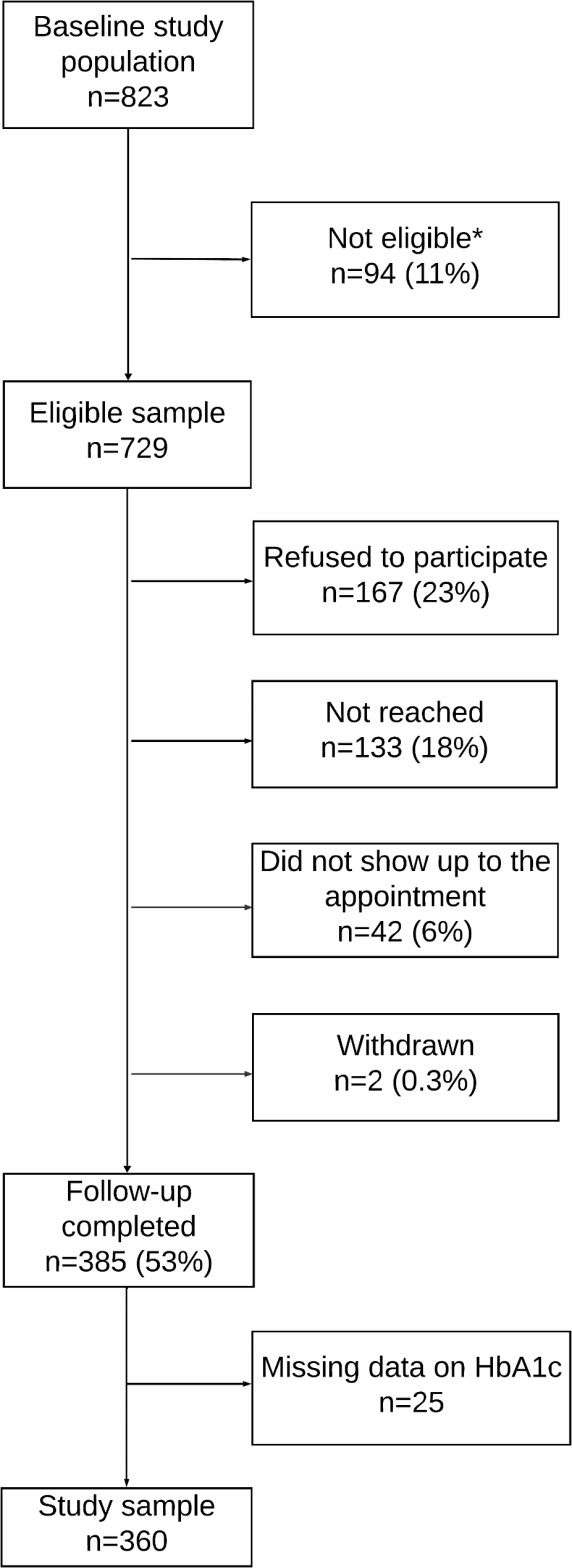
Flow chart. HbA1c based on capillary dried blood spot (*n* = 360, 94%)

Eleven years postpartum, 729 women (86% of the original cohort) still living in Norway and not excluded for reasons given in the flow chart (Fig. 1) were eligible for a follow-up (visit 4), 385 (53%) met and 360 (49.4%) had a valid HbA1c [14] and were included in the present study. All eligible women living in the Oslo region were invited by telephone to a new visit at the same study sites, including an interviewer-administered digital questionnaire, clinical measurements, and non-fasting capillary dried blood spots were sampled and biobanked [14]. In addition, women were asked to attend another day for a fasting blood sample. Recruitment of study participants stopped temporarily during three periods during the COVID-19 pandemic. Eligible women who had moved outside the area (*n* = 96, 95 ethnic Norwegians), were invited to a less extensive data collection based on telephone interviews. These women were encouraged to take a dried blood spot themselves, supported by oral and written information, but not asked to visit for fasting samples due to long travel distances [14].

Body height (cm) was measured using a fixed stadiometer. Body weight (kg) and fat mass (kg) were measured with bioimpedance analysis using the Tanita-MC-780MA. Actual parity was categorised as primipara, para 2 or para 3+. Information on present educational status was categorised as primary school, high school or college/university. HbA1c was measured in dried blood spot samples by the Vitas laboratory (Vitas AS Oslo, Norway) [14]. One single dried blood spot punch for each woman was subjected to hemolysis for 30 min at room temperature using 600 µl of lysate buffer. Subsequently, an aliquot of the lysate was analyzed for HbA1c using ELISA. The absorbance was read at 660 nm using Spark 10 M microplate-reader from Tecan, Switzerland. The analytical coefficient of variance was < 3%. In addition, blood samples from women who agreed to meet fasting 1–7 days after the clinical examination (*n* = 213) were also analysed at a routine, high-volume, Clinical Chemistry Laboratory (Fürst Laboratory, Oslo), for FPG (ADVIA Glucose Hexokinase) and HbA1c (Tosoh G11 HPLC). The HbA1c measurements performed on filter-paper and by HPLC showed good agreement [14].

### Power calculations

The original power calculations estimated that a sample size of 400 would provide a power of > 90% to detect significant differences for diabetes (2% vs. 8% in Europeans and South Asians, respectively) and prediabetes _WHO_ (FPG 6.1–6.9 mmol/L), 6% and 20%, respectively). Due to unforeseen restrictions (the COVID-19 pandemic and lack of funding), the number included in the present study was somewhat smaller.

### Statistical analyses

We summarized continuous data using the mean and standard deviation (SD) and compared mean differences between groups using the independent samples t-test. Categorical variables were described by frequencies and proportions and compared using the Chi-square test. We estimated relative risks (RR) using the generalized linear regression model with *binomial* family and a *log* link function.

We first performed univariate logistic regression analyses to identify potential predictors for our primary endpoint diabetes or prediabetes_ADA_. Thereafter, we used the least absolute shrinkage and selector operator (LASSO) which selected a subset of the best predictors, by shrinking the regression coefficients of the least contributing independent variables to zero. This implies that all the independent variables that were set to zero were eliminated from the predictive model. To achieve this, a tuning parameter lambda, which determines the amount of shrinkage was selected using 10-fold cross validation.

Identified input variables were:

- from visit 1: age, ethnicity, parity, education level, body height (cm), body weight (kg), pre-pregnant BMI (kg/m²), BMI (kg/m²), total fat mass (kg), HbA1c (mmol/mol), FPG (mmol/L), HOMA-IR, HOMA-β, triglycerides (mmol/L), Leptin (ng/ml) and first-degree relatives with diabetes.
- from visit 2: body weight (kg), BMI (kg/m²), total fat mass (kg), Leptin (ng/ml), HbA1c (mmol/mol), FPG (mmol/L), HOMA-IR, HOMA-β, 2-hour plasma glucose (mmol/L) and GDM_WHO2013_.
- from visit 3: body weight (kg), BMI (kg/m²), total fat mass (kg), triglycerides (mmol/L), Leptin (ng/ml), FPG (mmol/L), HOMA-IR and HOMA-β.

Due to some missing data particularly from the postpartum visit, we imputed missing data using the miceadds package in R and generated 10 different data sets, which we exported into Stata. We used predictive mean matching (pmm) to impute continuous variables, binary logistic regression (logreg) to impute binary variables and polytomous logistic regression (polyreg) to impute unordered factors. For each data set, a LASSO logistic regression model was fitted to the data. We compared the performances of the 10 fitted models and selected the model that included predictors that were also selected by the other nine LASSO models. More details about Model development and prediction of prediabetes or diabetes can be found in the Supplementary Box 1. However, since we did not have an external validation cohort to validate the prediction model, we considered a random sampling of the participants “with replacement” using bootstrapping. We used 500 bootstrap re-samplings.

The ability of the final predictive model in differentiating women with diabetes or prediabetes from those without was assessed using the area under the receiver operating characteristic curve (AUC). Threshold values of AUC ranging from 50 to 69% indicates that the ability of the model to differentiate women with diabetes or prediabetes from those without is poor, an AUC of 70–80% represents an acceptable model, 80–90% an excellent model and above 90% a model with outstanding performance.

**Fig. 2:**
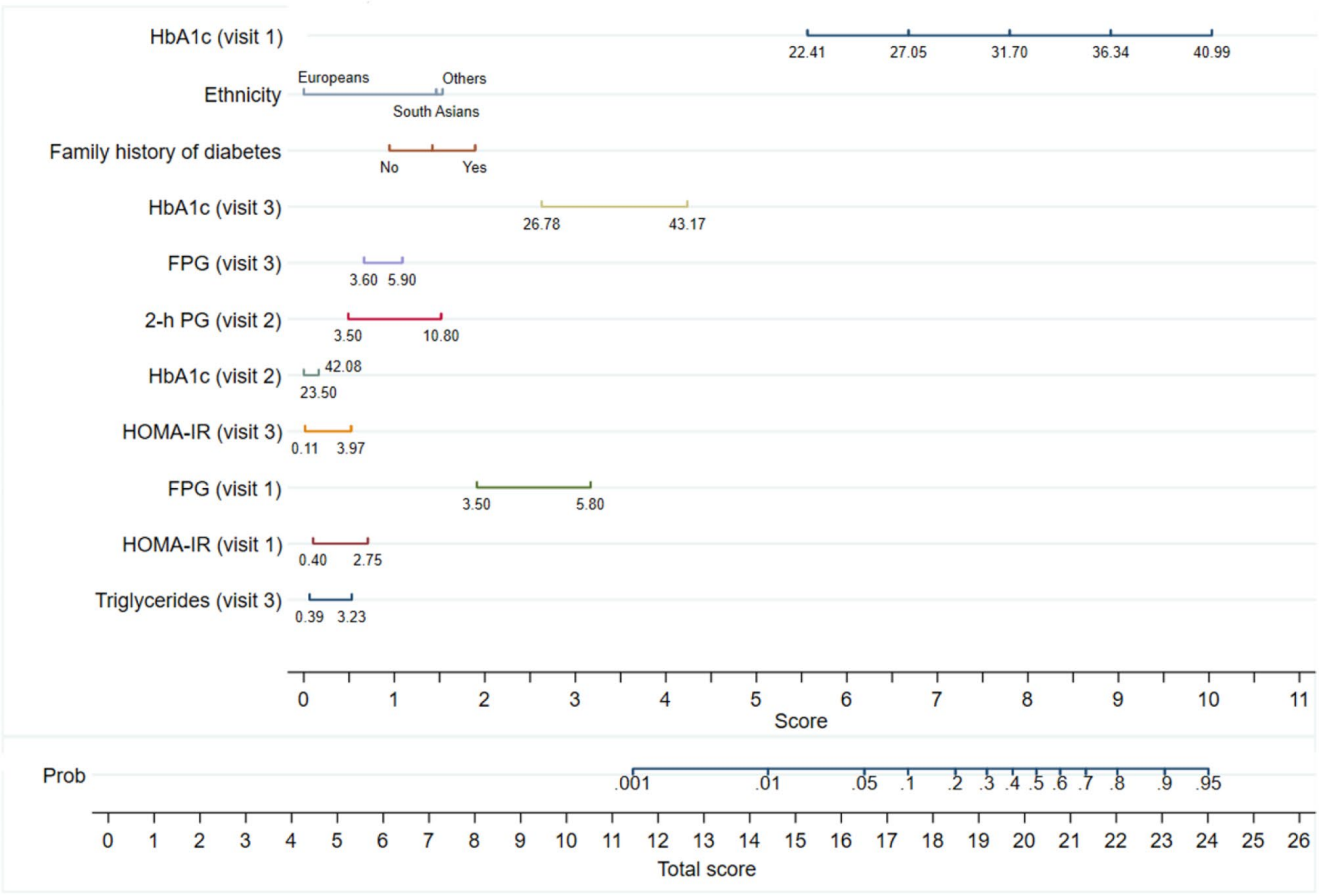
Nomogram estimating the probability of having prediabetes or diabetes as a function of 11 predictors. The predictors were selected by the Lasso logistic regression model. For a specific individual, each predictor value is given a score and a total score obtained by adding the individual scores. The total score is projected on total points axis to obtain the probability of prediabetes or diabetes

Finally, we developed a nomogram (Fig. 2, Supplementary Fig. 1a and 1b) based on the predictors selected by the LASSO logistic regression model to identify the risk for diabetes or prediabetes in individual women. The probability can be calculated as follows: scores for all predictors are obtained, summed to obtain a total score, which is then mapped on the probability graph.

All statistical analyses were performed in StataSE 18. A two-sided *P*-value < 0.05 was considered statistically significant (unless otherwise specified).

## Results

### Sample characteristics

Compared to women in the original cohort who did not meet at the eleven years follow-up, the 360 women included in the present study were somewhat older, more educated, and less likely to be from ethnic minorities (Supplementary Table 1). However, there were no differences in mean HbA1c or BMI between the two groups. The proportion of women fulfilling the GDM_WHO2013_ criteria in the index pregnancy was 28.9% among those included and 32.8% among those who did not meet for follow-up (*p* = 0.254).

Overall, 206 (57.2%) of the participants were of European ethnic background, 86 (23.9%) of South Asian and 68 (18.9%) of other ethnic backgrounds. Of the 360 included at the 11 years follow up study, mean age was 41.9 (SD 4.8), mean BMI was 27.0 (5.0) kg/m^2^ and 63.2% of the women had higher education (College/University). Hypertension (systolic blood pressure ≥ 140 mmHg or diastolic blood pressure ≥ 90 mmHg [21]) was found in 11.9% and morbid obesity (BMI ≥ 40 kg/m^2^) was observed in 1.4% of participants. Further characteristics for participants with and without GDM_WHO2013_ are shown in Table 1. At the baseline examination in gestational week 15, women with GDM were slightly older, more often had ancestral origin outside Europe, had higher parity and more often diabetes in their family. They had higher pre-pregnant and actual BMI, higher weight, HbA1c and FPG, compared with women without GDM. Also at the eleven years follow-up, actual BMI, HbA1c and FPG were higher in women with previous GDM than in those without (Table 1).

**Table 1:** Characteristics of participants with and without gestational diabetes (GDM) (WHO_2013_) in index pregnancy

		GDM *n* = 104 (28.9%)		No GDM *n* = 256 (71.1%)	*p* value
**Gestational week 15**	n		n		
Age at enrolment (years)	104	31.5 (4.8)	256	30.1 (4.5)	**0.007**
Parity (%)	104		256		**0.026**
Nulliparous	39	37.5	129	50.4	
Multiparous	65	62.5	127	49.6	
Education (%)	103		254		0.163
Primary school or less	12	11.6	21	8.3	
High school	43	41.8	87	34.2	
College/University	48	46.6	146	57.5	
Ethnicity (%)	104		256		**0.001**
Europe	45	43.3	161	62.9	
South Asia	37	35.6	49	19.1	
Other ethnicity	22	21.1	46	18.0	
Family history of type 2 diabetes (%)	101		253		**0.008**
Yes	30	29.7	43	17.0	
Pre-pregnant BMI (kg/m^2^)	104	25.9 (5.2)	253	23.8 (3.9)	**< 0.001**
Body height (cm)	104	163.7 (7.2)	256	164.4 (6.8)	0.419
Body weight (kg)	104	71.6 (15.3)	256	65.8 (12.0)	**< 0.001**
BMI (kg/m^2^)	104	26.7 (5.2)	256	24.3 (3.9)	**< 0.001**
HbA1c (mmol/mol)	102	33.9 (3.5)	250	32.7 (2.8)	**0.001**
HbA1c (%)	102	5.3 (0.3)	250	5.1 (0.3)	**0.001**
FPG (mmol/L)	103	4.7 (0.4)	253	4.3 (0.3)	**< 0.001**
**11 years follow up**					
Age at enrolment (years)	104	42.8 (4.9)	256	41.5 (4.7)	**0.022**
Time since index pregnancy (years)	102	10.8 (1.0)	254	10.9 (0.9)	0.104
Parity (%)	101		250		0.075
Primipara	8	7.9	27	10.8	
Para 2	43	42.6	132	52.8	
Para 3+	50	49.5	91	36.4	
Education (%)	102		254		0.149
Primary school	10	9.8	13	5.1	
High school	34	33.3	74	29.1	
College/University	58	56.9	167	65.8	
Body height (cm)	102	163.1 (7.4)	256	163.8 (6.9)	0.343
Body weight (kg)	104	75.2 (15.2)	254	70.9 (13.5)	**0.009**
BMI (kg/m^2^)	104	28.2 (5.3)	254	26.4 (4.9)	**0.002**
HbA1c (mmol/mol)	104	38.6 (6.1)	256	36.8 (3.2)	**< 0.001**
HbA1c (%)	104	5.7 (0.6)	256	5.5 (0.3)	**< 0.001**
FPG (mmol/L)	68	5.2 (0.9)	133	4.7 (0.6)	**< 0.001**

BMI: body mass index. GDM_2013_: FPG ≥ 5.1 or 2-hour PG ≥ 8.5 mmol/l. Other ethnicity: East Asia, Middle East, Africa. Participants were universally screened for GDM at mean gestational week 28. Values are presented as mean (sd) of frequencies (%). GDM versus no GDM were compared by two sample t-test or Pearson chi-squared test. P values < 0.05 are in bold

### Diabetes and prediabetes at follow-up

**Fig. 3:**
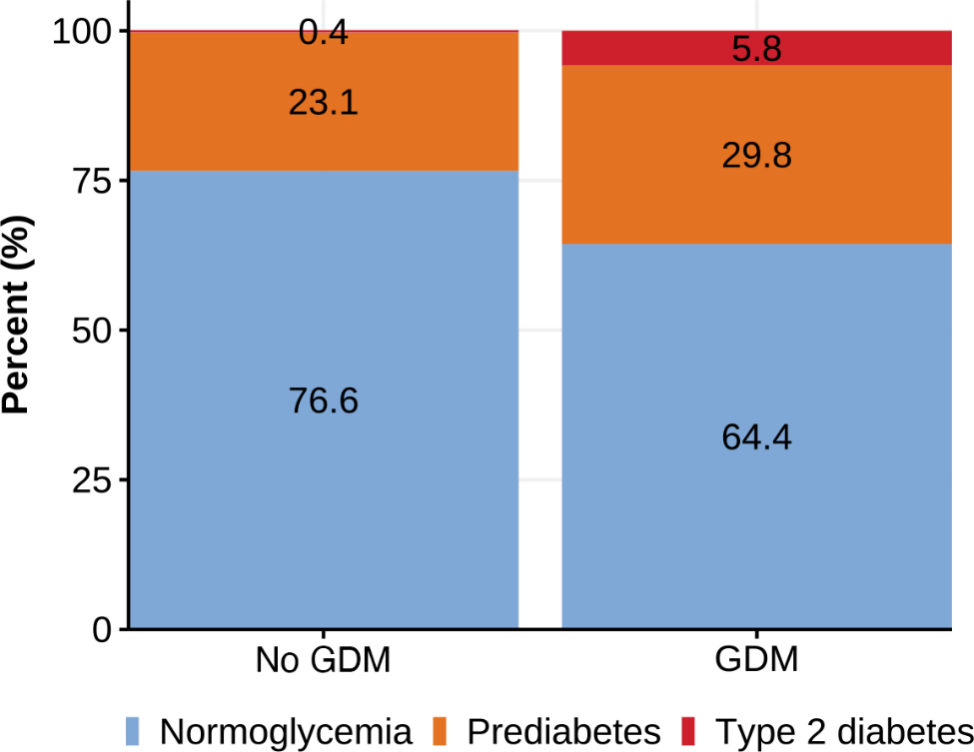
Prediabetes and diabetes by GDM (WHO_2013_) status

Diabetes or prediabetes_ADA_ combined (our primary outcome) was found in 26.9%, and the prevalence was slightly higher in women with GDM_WHO2013_ compared with women without GDM in the index pregnancy (35.6% versus 23.5%; *p* = 0.019) (Fig. 3, Supplementary Table 3). The RR for developing diabetes or prediabetes_ADA_ was moderately elevated in women with GDM in the index pregnancy compared to those without 1.4 (95% CI: 1.0, 1.9), *p* = 0.035.

Four women reported to have been diagnosed with diabetes prior to the study follow-up (two were treated with glucose lowering drugs). In addition, four women were detected with undiagnosed diabetes based on the HbA1c measured at the study visit. As one European woman with self-reported diabetes had a normal HbA1c (34 mmol/mol) without using glucose lowering drugs (since the postpartum visit she had 15 kg weight loss), a total of seven women (1.9%) had diabetes based on HbA1c, all with non-European ethnic origin (Supplementary Table 3). With a single exception, diabetes was only found in women with GDM_WHO2013_ in the index pregnancy, representing a prevalence of 5.8% GDM women (Supplementary Table 3) compared with 0.4% in non-GDM women. The RR for developing diabetes was substantially higher in GDM_WHO2013_ vs. non-GDM women 14.8 (95% CI: 2.6, 277.1), *p* = 0.012.

Prediabetes_ADA_ was found in 90 (25%) of the women (Supplementary Table 3) and the RR for developing prediabetes_ADA_ for GDM_WHO2013_ compared to non-GDM women was not significantly increased 1.3 (95% CI: 0.9, 1.8), *p* = 0.143. Using the more conservative definition of prediabetes as recommended by the WHO, only 24 women (6.7%) had prediabetes_WHO_, with a RR for of 3.2 (95% CI 1.6, 6.5), *p* = 0.001 in GDM vs. non-GDM women.

**Fig. 4:**
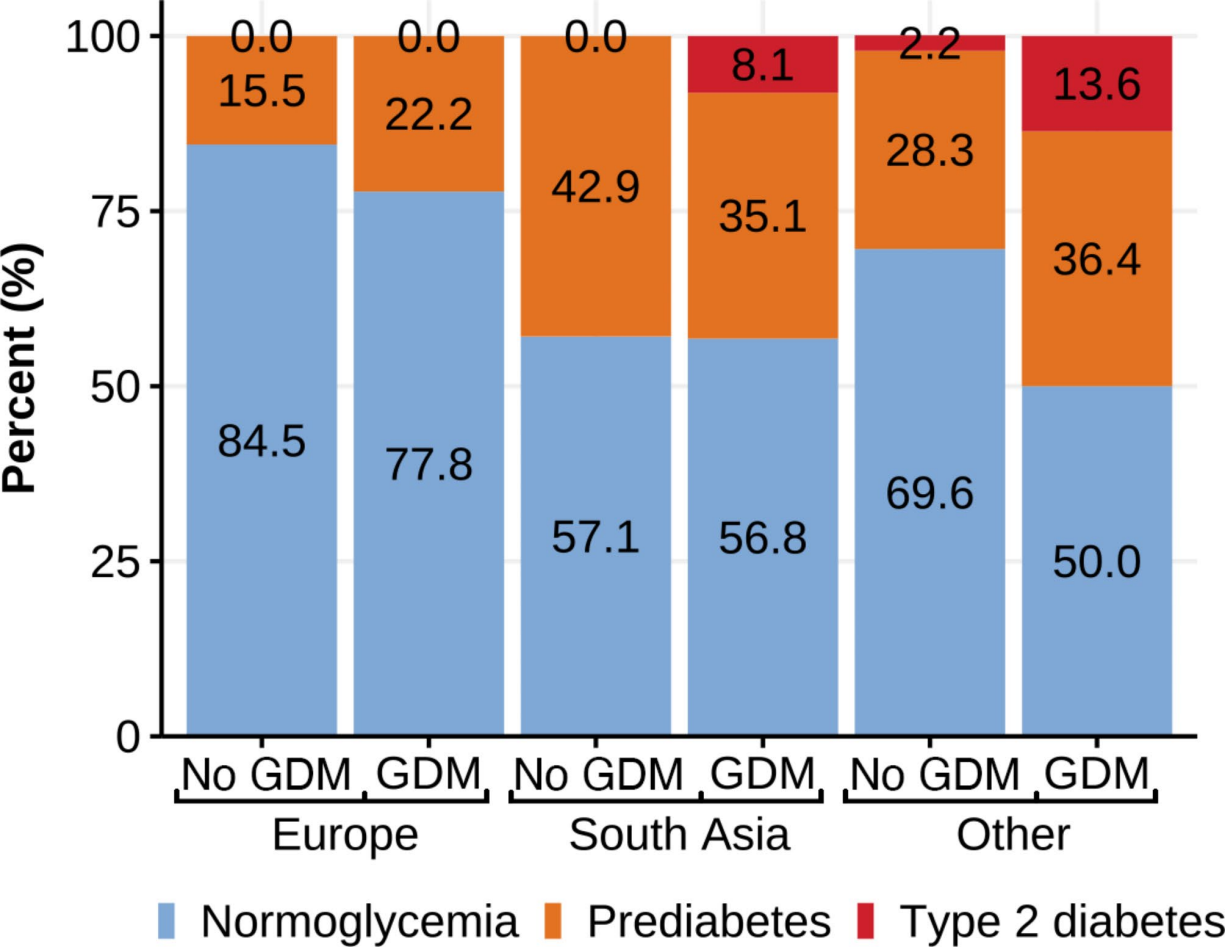
Prediabetes and diabetes by GDM (WHO_2013_) status by ethnic group

When investigating each ethnic group separately, 17.0% of women with European ethnicity had diabetes or prediabetes_ADA_ at follow-up, compared to 43.0% of South Asians (*p* < 0.001) and 34.4% of women from other ethnicities (*p* = 0.002) (Supplementary Table 3). The proportions with diabetes or prediabetes_ADA_ among GDM_WHO2013_ and non-GDM women are shown in Fig. 4. We found no difference in the prevalence of diabetes or prediabetes_ADA_ combined between the South Asian and the other ethnic minority groups, but European women had a substantially lower RR compared to the two ethnic minority groups combined (0.4 [0.3, 0.6], *p* < 0.001).

When applying the WHO_1999_ diagnostic criteria for GDM in the index pregnancy, only 45 (12.5%) had GDM. In these women, 12 (24.4%) had prediabetes_ADA_ at follow-up, while 4 (8.9%) had diabetes, compared with 3 (0.95%) in non-GDM women (Supplementary Table 3).

### Predictors for diabetes or prediabetes_ADA_ combined

**Fig. 5:**
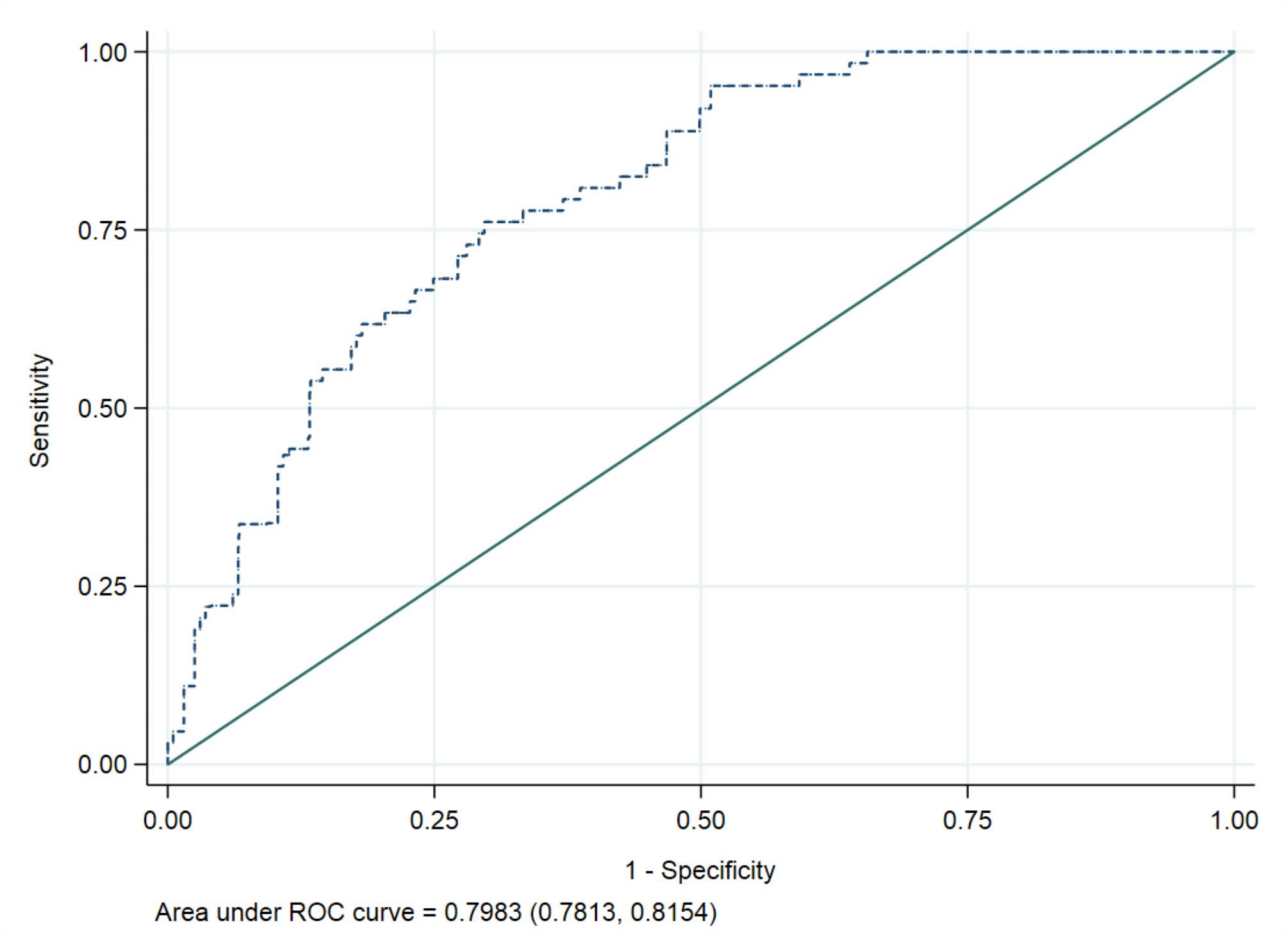
ROC curve from LASSO for the performance of the least absolute shrinkage and selection operator risk score in identifying women with increased risk for prediabetes and diabetes. The AUC under the ROC curve is 0.7983 (95% CI 0.7813, 0.8154)

**Fig. 6:**
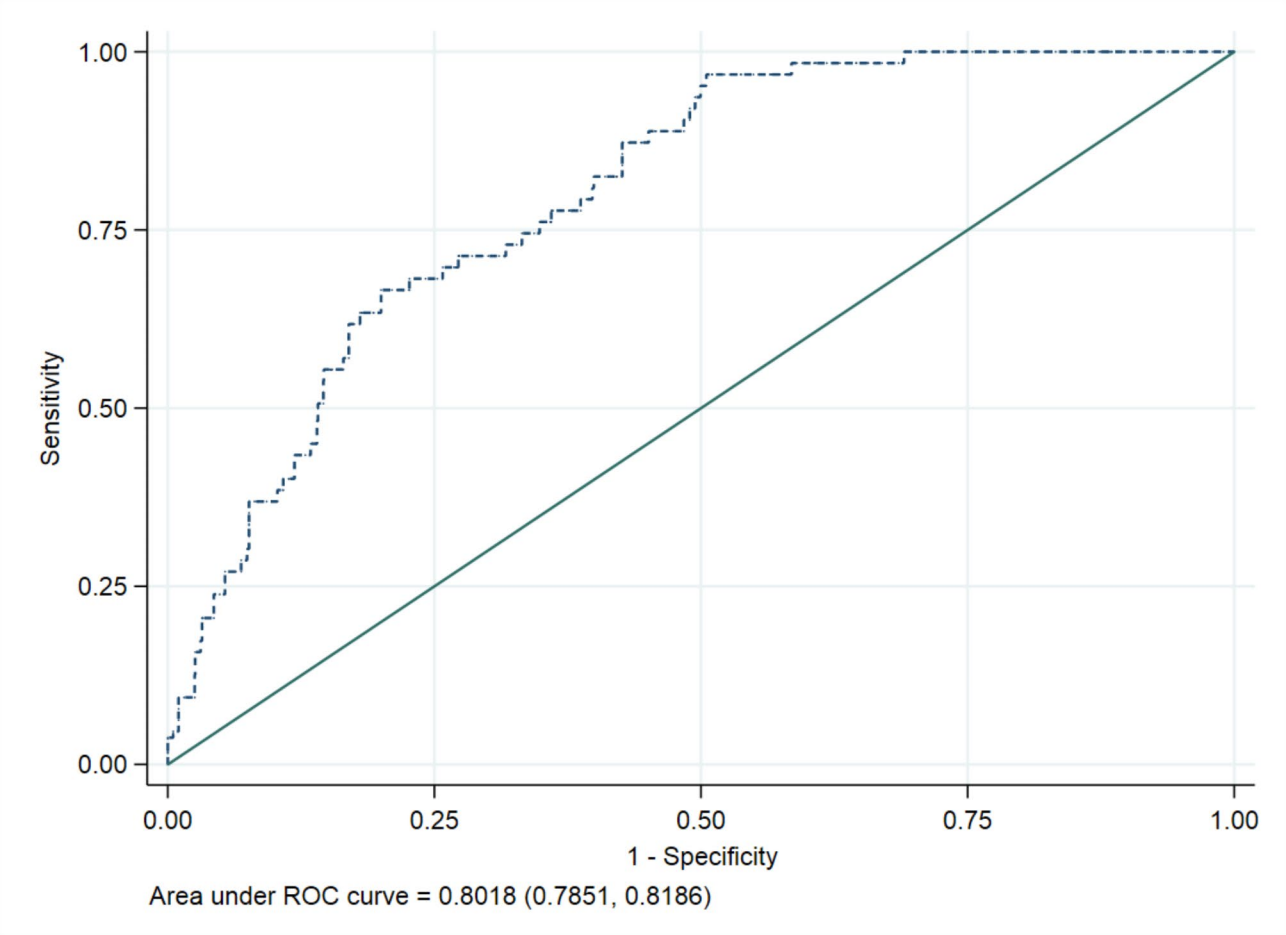
ROC curve from Nomogram for the performance of the least absolute shrinkage and selection operator risk score in identifying women with increased risk for prediabetes and diabetes. The AUC under the ROC curve is 0.8018 (95% CI 0.7831, 0.8186)

Supplementary Table 4 shows the relationship between potential predictors from visit 1 to 3 and the risk of developing diabetes or prediabetes _ADA_ as observed. After analysing these factors with LASSO, the most significant predictors identified were HbA1c, ethnicity and a family history of diabetes, all recorded in early pregnancy (Table 2). Additionally, fasting FPG and HOMA-IR, also measured at 15 weeks of pregnancy, along with HbA1c, FPG, HOMA-IR, and triglycerides measured postpartum, were important predictors. While GDM_WHO2013_ itself was not a direct predictor, HbA1c in early pregnancy and the 2-hour glucose levels from the OGTT at 28 weeks of pregnancy were (Table 2). The model’s AUC was 0.80 (95% CI 0.78, 0.82), indicating a high accuracy in predicting the combined risk of our primary outcome (Fig. 5). Internal validation using bootstrapping confirmed the model’s good predictive performance with an AUC of 0.80 (95% CI 0.79, 0.82) (Fig. 6). A nomogram for probability of developing diabetes or prediabetes_ADA_ is presented in Fig. 2. The predictive performance of the nomogram gave an estimated C-index of ≥ 0.7. Examples of calculated risks for two participating women with different ethnicity are shown in Supplementary Fig. 1a and 1b.

**Table 2:** LASSO^a^ prediction model for prediabetes and diabetes after 11 years^b^

Predictors selected from visit 1–3	LASSO coefficient
Constant	-1.303
HbA1c (Visit 1)	0.424
Ethnicity (European)	-0.422
Family history of diabetes	0.152
HbA1c (Visit 3)	0.087
FPG (Visit 3)	0.062
2-hour PG (Visit 2)	0.043
HbA1c (Visit 2)	0.037
HOMA-IR (Visit 1)	0.037
FPG (Visit 1)	0.023
HOMA-IR (Visit 3)	0.019
Triglycerides (Visit 3)	0.015

^a^LASSO: least absolute shrinkage and selection operator

^b^Dysglycemia: prediabetes 39–47 mmol/mol and diabetes ≥ 48 mmol/mol

Visit 1: gestational week 15. Visit 2: gestational week 28. Visit 3: 14 weeks postpartum

Variables not selected by the model: education, age, parity, glucose (Visit 2),

Pre-pregnant BMI, BMI (Visit 1–3), triglycerides (Visit 1 and 2), HOMA-IR (Visit 2),

HOMA-β (Visit 1–3), Leptin (Visit 1–3), total fat mass (Visit 1–3), GDM_2013_

## Discussion

In this prospective, population-based cohort study of a multi-ethnic population of women universally screened for GDM_WHO2013_, we found that one in three with GDM in the index pregnancy had the combined primary outcome diabetes or prediabetes_ADA_ at the follow-up visit. We observed a high prevalence of prediabetes among ethnic minorities with women of South Asian background at highest risk. Important predictors for dysglycemia were HbA1c level from early pregnancy, ethnic minority background and family history of diabetes.

Only very few of these women with a mean age of 42 years at follow-up had developed type 2 diabetes. We observed a slightly higher prevalence of dysglycemia in women with previous GDM. However, the RR for developing diabetes was substantially higher in previous GDM vs. non-GDM women.

### Results in context – what’s new

This is one of few long-term follow-up studies of a population-based, multi-ethnic cohort, universally screened for GDM, showing the long-term consequences for the mother of being diagnosed with GDM by the new WHO_2013_ criteria and the older WHO_1999_ criteria. In the total cohort, we have previously reported a GDM prevalence of 13.0% by the WHO_1999_ criteria [17], but most cases were mild, as only five were treated with glucose lowering drugs in pregnancy. The prevalence of GDM was, however, substantially higher (31.5%) by the WHO_2013_ criteria [17]. Those who met the WHO_2013_ criteria, but not diagnosed by the WHO_1999_ criteria and therefore not treated, had also mostly a mild GDM (a moderately elevated FPG and a 2-hour value < 7.8 mmol/l). Still, in a larger study where four Norwegian studies were pooled, this group had an increased risk of large for gestational age neonates, caesarean section and operative vaginal delivery compared to women without GDM [22].

Although the prevalence of prediabetes_ADA_ was in line with other reports [23], the prevalence of diabetes was lower overall (1.9%) than estimated in our pre-specified power calculations, and in women with previous GDM (5.8%), substantially lower than reported in most previous studies (up to > 50%) [24] and a recent meta-analysis [5]. Our results are, however, in line with a German study [25], which reported a diabetes prevalence of 6% after five years among women with previous GDM_WHO2013_. We suggest that the major reason is that GDM detected by universal screening in a non-selected population identifies a group of women that differ from those included in many observational studies of GDM, that usually are based on referrals to secondary health care or on women that were subject to targeted screening in high-risk populations. Further, many studies have used OGTT-diagnosed diabetes as the outcome, while we used HbA1c that is the recommended diagnostic test for diabetes today. The prevalence of diabetes diagnosed by HbA1c is generally lower than based on OGTT [26]. However, importantly, in the present study, one in three women with GDM in the index pregnancy identified with both the WHO_1999_ and the WHO_2013_ criteria had the primary outcome after universal screening, and GDM, particularly by the WHO_2013_ criteria represented a strong risk for diabetes, in line with other studies.

On the other hand, at follow-up, irrespective of the GDM definition used, GDM_WHO2013_ constituted only a modest risk for the combined outcome (RR 1.4–3.6) and for prediabetes dependent on the definition (RR 1.3–3.2). Importantly, HbA1c at 15 weeks of pregnancy outperformed GDM diagnosed at gestational week 28 as predictor of the primary outcome in the LASSO regression model.

Few previous studies have such a rich dataset of potential predictors from pre-pregnancy and early pregnancy. The ethnic differences in diabetes and prediabetes_ADA_ at the follow-up visit confirm previous findings. However, with information about family history of diabetes and ethnicity, HbA1c values from gestational week 15 can be used as a strong predictor for later diabetes or prediabetes_ADA_. Other measures of glucose and HbA1c during later pregnancy and postpartum were also relevant and may reflect the effects of pregnancy more precisely than a diagnosis of GDM per se. Of note, no measures of baseline adiposity were included in the LASSO prediction model, probably because these factors are mediated by HbA1c and ethnicity already captured in the model.

### Clinical relevance

As almost all pregnant women meet health care professional during pregnancy, they represent a unique potential for relevant health information and suggestions and support for prevention strategies. We find that simple clinical information like ethnic background and family history of diabetes combined with a single measurement of HbA1c early in pregnancy may provide the clinician a tool for relevant risk stratification regarding future diabetes. In this follow up study we chose to diagnose diabetes or prediabetes using only HbA1c and not an OGTT. This is in accordance with the recommendations in the Norwegian national guidelines for regular follow-up after GDM and also approved by the ADA [27].

### Strengths and limitations

An important strength of the present study is the long-term follow-up of a cohort in an age-group highly relevant for preventive strategies. Further strengths include the population-based approach in a multi-ethnic population, also including “hard to reach” women (about 22% needed a translator at inclusion). We consider that a 53% (49% with HbA1c measured) attendance rate at the eleven years follow-up visit despite the ongoing COVID-19 epidemic was decent, although obviously we planned for a higher participation rate. Universal screening with OGTT allowed for reliable estimates of GDM with different diagnostic criteria and the detailed data collected during and after pregnancy provided the opportunity for developing prediction models. To account for missing data, particularly at the postpartum visit, we performed multiple imputations to reduce the risk of biased estimates. The classification of diabetes or prediabetes as outcomes was based on HbA1c, which is the common measure used by clinicians today. However, HbA1c values in pregnancy must be interpreted with caution, as the levels are altered independent of glycaemia, due to changes in the red-cell turnover rate [28]. Further, ethnic background may influence HbA1c levels, as people of European decent have been reported to have lower HbA1c levels compared to other ethnic groups, e.g., African Americans and Asian Americans despite similar plasma glucose levels [29, 30].

Limitations include the fact that we were only able to recruit about half of the original cohort to this follow-up study, resulting in a relatively small sample size. Further, the number of individuals that developed diabetes was low, limiting the power for subgroup analyses. Further, as no OGTTs were performed at the eleven years visit, we cannot compare HbA1c-diagnosed and OGTT-diagnosed diabetes prevalence and how the prediction model would work for OGTT-diagnosed outcomes.

## Conclusions

The prevalence of diabetes was low, both overall and among women with previous GDM. Still, GDM was a strong risk factor for diabetes, but not so for our primary combined endpoint of diabetes and prediabetes_ADA_. Non-European ethnicity, a family history of diabetes and HbA1c early in pregnancy were the strongest risk factors for developing diabetes or prediabetes_ADA_, as determined using an artificial intelligence algorithm.

## Electronic supplementary material

Below is the link to the electronic supplementary material.

Supplementary Material 1

## Data Availability

The editors can access data (in de-identified form) used in the manuscript, code book, and analytical code upon request. The project manager and the head of our department will contribute to the access being provided under appropriate conditions. However, research data for this publication include identifying health information subject to confidentiality. It is therefore not possible to share raw data.

## Abbreviations

GDM: Gestational diabetes mellitus
OGTT: Oral glucose tolerance test
STORK G: Stork Groruddalen
HbA1c: Glycated haemoglobin
FPG: Fasting plasma glucose
HOMA-IR: Homeostatic model assessment for insulin resistance
HOMA-β: Homeostatic model assessment for beta-cell function
BMI: Body mass index
SD: Standard deviation
OR: Odds ratio
CI: Confidence interval
RR: Relative risk
LASSO: Least absolute shrinkage and selector operator
WHO_2013_: World Health Organization criteria for diagnosing gestational diabetes, updated 2013
WHO_1999_: World Health Organization criteria for diagnosing gestational diabetes from 1999
