# Supplementary material for: Diabetes and prediabetes among women universally screened for gestational diabetes: a multi-ethnic, population-based, prospective study with eleven years follow-up
Source: BMC Public Health. 2025 Apr 3;25:1264. doi: 10.1186/s12889-025-22493-x (PMC11969744; doi:10.1186/s12889-025-22493-x)
Supplement: Supplementary file 1 — Supplementary Material 1 [file 12889_2025_22493_MOESM1_ESM.docx]

Diabetes and prediabetes among women universally screened for gestational diabetes: a multi-ethnic, population-based, prospective study with eleven years follow-up

Christin W. Waage^ab^, Anne Karen Jenum^a^, Ibrahimu Mdala^a^, Sindre Lee-Ødegård^cd^, Anja Maria Brænd^a^, Line Sletner^a^, Jens Petter Berg^ce^, Kåre I. Birkeland^cd^

**Affiliations**

^a^General Practice Research Unit (AFE), Department of General Practice, Institute of Health and Society, University of Oslo, Oslo Norway.

ᵇDepartment of Rehabilitation Science and Health Technology, Faculty of Health Sciences, Oslo Metropolitan University, Oslo Norway.

^c^Institute of Clinical Medicine, Faculty of Medicine, University of Oslo, Oslo Norway.

^d^Department of Endocrinology, Obesity and Preventive Medicine, Oslo University Hospital, Oslo Norway.

^e^Department of Medical Biochemistry, Oslo University Hospital, Oslo Norway

**Correspondence** Christin W. Waage, General Practice Research Unit (AFE), Department of General Practice, University of Oslo, Institute of Health and Society, Post Box 1130 Blindern, N-0318 Oslo, Norway. Mobile: +47 93243842. E-mail: [c.w.waage@medisin.uio.no](mailto:c.w.waage@medisin.uio.no).

**Supplementary table 1.** Characteristics of the cohort at mean gestational week 15

|  | **Included**  n=360 (49.4) | | **Not included**  n=369 (50.6) | | **p value** |
| --- | --- | --- | --- | --- | --- |
|  | n |  | n |  |  |
| Age (years) | 360 | 30.5 (4.7) | 369 | 29.5 (4.9) | **0.004** |
| BMI (kg/m²) | 360 | 24.9 (4.4) | 364 | 25.7 (5.3) | 0.068 |
| HbA1c (mmol/mol) | 352 | 33.1 (3.1) | 363 | 32.9 (3.3) | 0.738 |
| HbA1c (%) | 352 | 5.2 (0.3) | 363 | 5.2 (0.3) | 0.738 |
| Education (%) | 357 |  | 367 |  | **<0.001** |
| Primary school or less | 33 | 9.2 | 79 | 21.5 |  |
| High school | 130 | 36.4 | 149 | 40.6 |  |
| College/university | 194 | 54.3 | 139 | 37.9 |  |
| Parity (%) | 360 |  | 369 |  | 0.649 |
| Nullipara | 168 | 46.7 | 166 | 45.0 |  |
| Multipara | 192 | 53.3 | 203 | 55.0 |  |
| Ethnicity (%) | 360 |  | 369 |  | **<0.001** |
| Europe | 206 | 57.2 | 141 | 38.2 |  |
| South Asia | 86 | 23.9 | 95 | 25.8 |  |
| Other ethnicityᵃ | 68 | 18.9 | 133 | 36.0 |  |
| GDM (WHO_2013_) | 104 | 28.9 | 121 | 32.8 | 0.254 |

Eligible sample (n=729) stratified by included and those not included at the 11-years follow-up. Values are presented as mean (sd) of frequencies (%). Other ethnicity: East Asia, Middle East, Africa.

**Supplementary table 2.** Characteristics of participants with and without gestational diabetes (GDM_WHO1999_) in index pregnancy

|  |  | **GDM**  n=45 (12.5%) |  | **No GDM**  n=315 (87.5%) | **p value** |
| --- | --- | --- | --- | --- | --- |
| **Gestational week 15** | n |  | n |  |  |
| Age at enrolment (years) | 45 | 32.5 (4.7) | 315 | 30.2 (4.6) | **0.026** |
| Parity (%) |  |  |  |  | 0.523 |
| Nullipara | 19 | 42.2 | 149 | 47.3 |  |
| Multipara | 26 | 57.8 | 166 | 52.7 |  |
| Education (%) |  |  |  |  | 0.079 |
| Primary school or less | 8 | 18.2 | 25 | 8.0 |  |
| High school | 16 | 36.4 | 114 | 36.4 |  |
| College/University | 20 | 45.4 | 174 | 55.6 |  |
| Ethnicity (%) |  |  |  |  | 0.161 |
| Europe | 20 | 44.4 | 186 | 59.0 |  |
| South Asia | 13 | 28.9 | 73 | 23.2 |  |
| Other ethnicity | 12 | 26.7 | 56 | 17.8 |  |
| Family history of type 2 diabetes (%) | 45 |  | 309 |  | **0.024** |
| Yes | 15 | 33.3 | 58 | 18.8 |  |
| Pre-pregnant BMI (kg/m^2^) | 45 | 25.7 (4.7) | 312 | 24.2 (4.3) | **0.031** |
| Body height (cm) | 45 | 160.0 (6.9) | 315 | 164.8 (6.8) | **<0.001** |
| Body weight (kg) | 45 | 67.5 (13.3) | 315 | 67.2 (13.2) | 0.868 |
| BMI (kg/m^2^) | 45 | 26.2 (4.7) | 315 | 24.8 (4.4) | 0.051 |
| HbA1c (mmol/mol) | 45 | 33.4 (3.4) | 307 | 33.0 (3.0) | 0.494 |
| HbA1c (%) | 45 | 5.2 (0.3) | 307 | 5.2 (0.3) | 0.494 |
| FPG (mmol/L) | 44 | 4.6 (0.4) | 312 | 4.4 (0.4) | **0.002** |
| **11 years follow up** |  |  |  |  |  |
| Age at enrolment (years) | 45 | 43.8 (4.8) | 315 | 41.6 (4.7) | **0.004** |
| Time since index pregnancy (years) | 43 | 10.9 (0.8) | 313 | 10.9 (0.9) | 0.955 |
| Parity (%) |  |  |  |  | 0.519 |
| Primipara | 4 | 9.5 | 31 | 10.0 |  |
| Para 2 | 19 | 45.2 | 156 | 50.5 |  |
| Para 3+ | 19 | 45.2 | 122 | 39.5 |  |
| Education (%) |  |  |  |  | **0.028** |
| Primary school | 7 | 16.3 | 16 | 5.1 |  |
| High school | 13 | 30.2 | 95 | 30.4 |  |
| College/University | 23 | 53.5 | 202 | 64.5 |  |
| Body height (cm) | 45 | 159.4 (7.1) | 315 | 164.2 (6.9) | **<0.001** |
| Body weight (kg) | 45 | 71.2 (13.5) | 313 | 72.3 (14.2) | 0.63 |
| BMI (kg/m^2^) | 45 | 28.1 (5.4) | 313 | 26.8 (4.9) | 0.115 |
| HbA1c (mmol/mol) | 45 | 38.7 (5.8) | 315 | 37.1 (4.0) | **0.026** |
| HbA1c (%) | 45 | 5.7 (0.5) | 315 | 5.6 (0.4) | **0.026** |
| FPG (mmol/L) | 33 | 5.2 (1.1) | 168 | 4.8 (0.7) | **0.009** |

BMI: body mass index. GDM by the WHO_1999_ criteria: FPG ≥7.0 or 2-hour glucose ≥7.8 mmol/L. Other ethnicity: East Asia, Middle East, Africa. Participants were universally screened for GDM at mean gestational week 28. Values are presented as mean (sd) of frequencies (%). GDM versus no GDM were compared by two sample t-test for continues variables or Pearson chi-squared test for categorical variables. P values <0.05 are in bold.

**Supplementary table 3.** Prevalence of prediabetes and diabetes 11-years after index pregnancy by gestational diabetes

|  | **Total** | | | | | | **Europe** | | | | | | **South Asia** | | | | | | **Other ethnicity** | | | | | |
| --- | --- | --- | --- | --- | --- | --- | --- | --- | --- | --- | --- | --- | --- | --- | --- | --- | --- | --- | --- | --- | --- | --- | --- | --- |
|  |  |  | **GDM WHO_1999_** | | **GDM WHO_2013_** | |  |  | **GDM WHO_1999_** | | **GDM WHO_2013_** | |  |  | **GDM WHO_1999_** | | **GDM WHO_2013_** | |  |  | **GDM WHO_1999_** | | **GDM WHO_2013_** | |
|  | n | % | n | % | n | % | n | % | n | % | n | % | n | % | n | % | n | % | n | % | n | % | n | % |
| Total | 360 |  | 45 | 12.5 | 104 | 28.9 | 206 |  | 20 | 9.7 | 45 | 21.8 | 86 |  | 13 | 15.1 | 37 | 43 | 68 |  | 12 | 17.6 | 22 | 32.4 |
| Normal | 263 | 73.1 | 30 | 66.7 | 67 | 64.4 | 171 | 83.0 | 15 | 75.0 | 35 | 77.8 | 49 | 57.0 | 7 | 53.9 | 21 | 56.8 | 43 | 63.2 | 8 | 66.7 | 11 | 50.0 |
| Prediabetes (38-47 mmol/mol) (ADA) | 90 | 25.0 | 11 | 24.4 | 31 | 29.8 | 35 | 17.0 | 5 | 25.0 | 10 | 22.2 | 34 | 39.5 | 4 | 30.8 | 13 | 35.1 | 21 | 30.9 | 2 | 16.7 | 8 | 36.4 |
| Diabetes (≥ 48 mmol/mol) | 7 | 1.9 | 4 | 8.9 | 6 | 5.8 | 0 | 0 | 0 | 0 | 0 | 0 | 3 | 3.5 | 2 | 15.4 | 3 | 8.1 | 4 | 3.5 | 2 | 16.7 | 3 | 13.6 |
| Prediabetes and diabetes | 97 | 26.9 | 15 | 33.3 | 37 | 35.6 | 35 | 17.0 | 5 | 25.0 | 10 | 22.2 | 37 | 43.0 | 6 | 46.2 | 16 | 43.2 | 25 | 34.4 | 4 | 33.3 | 11 | 50.0 |

GDM: Gestational diabetes.

ADA: American Diabetes Association. Other ethnicity: East Asia, Africa, Middle East. GDM by the WHO_1999_ criteria: FPG ≥7.0 or 2-hour glucose ≥7.8 mmol/L. GDM by the WHO_2013_ criteria: FPG ≥5.1 or 2-hour glucose ≥8.5 mmol/l.

**Supplementary table 4.** Potential predictors for prediabetes and diabetes 11-years after index pregnancy

|  | **Unadjusted** | | | | |
| --- | --- | --- | --- | --- | --- |
|  | **n** | **OR** | **(95% CI)** | | **P** |
| **Predictors from visit 1** |  |  | **Lower** | **Upper** |  |
| Age (years) | 360 | 1.03 | 0.97 | 1.08 | 0.283 |
| Ethnicity | 360 |  |  |  |  |
| Europe | Reference | | | | |
| South Asia |  | **3.69** | **2.11** | **6.46** | **<0.001** |
| Other |  | **2.84** | **1.54** | **5.24** | **0.001** |
| Parity |  |  |  |  |  |
| Nulliparous | Reference | | | | |
| Multiparous | 360 | 1.43 | 0.89 | 2.3 | 0.137 |
| Education (years) |  |  |  |  |  |
| ≥10 | Reference | | | | |
| <10 | 357 | 1.86 | 0.89 | 3.9 | 0.102 |
| Body height (cm) | 360 | 0.98 | 0.95 | 1.01 | 0.244 |
| Body weight (kg) | 360 | 1.01 | 0.99 | 1.03 | 0.355 |
| Pre-pregnancy BMI (kg/m²) | 357 | 1.04 | 0.99 | 1.0 | 0.106 |
| BMI (kg/m²) | 360 | 1.04 | 0.99 | 1.1 | 0.136 |
| Fat mass, total (kg) | 360 | 1.01 | 0.99 | 1.04 | 0.284 |
| Body height (cm) | 360 | 0.98 | 0.95 | 1.01 | 0.244 |
| Body weight (kg) | 360 | 1.01 | 0.99 | 1.03 | 0.355 |
| Pre-pregnancy BMI (kg/m²) | 357 | 1.04 | 0.99 | 1.0 | 0.106 |
| HbA1c (mmol/mol) | **352** | **1.41** | **1.27** | **1.57** | **<0.001** |
| FPG (mmol/L) | **356** | **2.99** | **1.66** | **5.40** | **<0.001** |
| Leptin (ng/ml) | 356 | 1.0 | 0.99 | 1.0 | 0.776 |
| HOMA-IR | **332** | **2.28** | **1.37** | **3.81** | **0.002** |
| HOMA-β | 332 | 1.0 | 0.99 | 1.01 | 0.300 |
| Triglycerides (mmol/L) | 359 | 1.47 | 0.96 | 2.23 | 0.074 |
| Family history of type 2 diabetes (yes) | **73** | **2.41** | **1.4** | **4.16** | **0.001** |
| **Predictors from visit 2** |  |  |  |  |  |
| Body weight (kg) | 356 | 1.03 | 0.98 | 1.09 | 0.276 |
| BMI (kg/m²) | 356 | 1.04 | 0.99 | 1.1 | 0.133 |
| Fat mass, total (kg) | 354 | 1.0 | 0.98 | 1.03 | 0.784 |
| HbA1c (mmol/mol) | **346** | **1.23** | **1.13** | **1.33** | **<0.001** |
| FPG (mmol/L) | **356** | **2.09** | **1.28** | **3.40** | **0.003** |
| 2-h PG (mmol/L) | **355** | **1.28** | **1.08** | **1.51** | **0.004** |
| Leptin (ng/ml) | 350 | 1.0 | 0.99 | 1.0 | 0.513 |
| HOMA-IR | 348 | 1.17 | 0.92 | 1.49 | 0.212 |
| HOMA-β | 348 | 0.99 | 0.99 | 1.00 | 0.441 |
| Triglycerides (mmol/L) | 355 | 1.38 | 0.98 | 1.94 | 0.62 |
| GDM_2013_ | 356 | 1.73 | 1.05 | 2.85 | **0.033** |
| Body weight (kg) | 356 | 1.03 | 0.98 | 1.09 | 0.276 |
| BMI (kg/m²) | 356 | 1.04 | 0.99 | 1.1 | 0.133 |
| **Predictors from visit 3** |  |  |  |  |  |
| Body weight (kg) | 326 | 1.01 | 0.99 | 1.03 | 0.136 |
| BMI (kg/m²) | 326 | 1.06 | 1.0 | 1.12 | **0.046** |
| Fat mass, total (kg) | 325 | 1.02 | 0.99 | 1.05 | 0.104 |
| Triglycerides (mmol/L) | **284** | **2.33** | **1.38** | **3.92** | **0.002** |
| HbA1c (mmol/mol) | **277** | **1.28** | **1.15** | **1.42** | **<0.001** |
| FPG (mmol/L) | **282** | **3.15** | **1.57** | **6.33** | **0.001** |
| Leptin (ng/ml) | **284** | **1.0** | **1.0** | **1.0** | **0.010** |
| HOMA-IR | **277** | **1.98** | **1.26** | **3.13** | **0.003** |
| HOMA-β | 277 | 1.0 | 0.99 | 1.0 | 0.286 |

*Prediabetes according to the American Diabetes Association (ADA) criteria. GDM by the WHO_2013_ criteria: FPG ≥5.1 or 2-hour PG ≥8.5 mmol/l. Visit 1: Gestational week 15. Visit 2: Gestational week 28. Visit 3: 14 weeks postpartum. Data are odds ratio (OR) 95% confidence interval (CI). Bold numbers indicate P-values <0.05. Statistics: logistic regression analysis.

**Supplementary Box 1:** **Model development and prediction of prediabetes and diabetes**

To classify whether a woman has prediabetes, diabetes or not, the lasso logistic regression classifier, which is a machine learning technique was used. Using the predictors selected by the lasso logistic regression model, we illustrate here how estimates of risk scores were obtained and used to predict prediabetes and diabetes.

- **Binary outcome:** dysglycemia (Yes/ No)
- **Predictor variables considered:**
  - **Visit 1:** ethnicity, education level, glucose, leptin, HOMA-β, HbA1c, age, parity, pre-pregnancy BMI, BMI, total fat, HOMA-IR, triglycerides, family history of diabetes.
  - **Visit 2:** BMI, total fat, triglycerides, leptin, HbA1c, Glucose, HOMA-IR, HOMA- β, 2-h PG.
  - **Visit 3:** GDM (WHO _2013_), BMI, total fat, triglycerides, HbA1c, glucose, leptin, HOMA-IR, HOMA- β.

The lasso regression classifier selected the following predictor variables: HbA1c (v1, v2, v3), ethnicity, family history of diabetes, glucose (v1, v3), 2-h PG (v2), HOMA-IR (v1, v3), HOMA- β (v1) and triglycerides (v3).

Based on the model, the probability of being classified as having prediabetes or diabetes using the selected predictors is calculated as follows:

exp (a woman’s risk score) [1$+$ exp (a woman’s risk score)]

Where a woman’s **risk score** is estimated as follows:

$\hat{\beta_{0}}$ - $\hat{\beta_{1}}$ HbA1c (V1)+ $\hat{\beta_{2}}$ ethnicity + $\hat{\beta_{3}}$ family history + $\hat{\beta_{4}}$ HbA1c (V3) + $\hat{\beta_{5}}$ glucose (V3) + $\hat{\beta_{6}}$ 2-h PG (V2) + $\hat{\beta_{7}}$ HbA1c (V2) + $\hat{\beta_{8}}$ HOMA-IR (V1) + $\hat{\beta_{9}}$ glucose (V1) + $\hat{\beta_{10}}$ HOMA-IR (V3)+ $\hat{\beta_{11}}$ triglycerides (V3)

Here, $\hat{\beta_{0}}$, $\hat{\beta_{1}}$, $\hat{\beta_{2}}$, $\hat{\beta_{3}}$, $\hat{\beta_{4}}$, $\hat{\beta_{5}}$, $\hat{\beta_{6}}$, $\hat{\beta_{7}}$, $\hat{\beta_{8}}$, $\hat{\beta_{9}}$, $\hat{\beta_{10}} a$nd $\hat{\beta_{11}}$ represent the lasso logistic regression coefficients corresponding to the intercept, HbA1c (v1), ethnicity, family history, HbA1c (v3), glucose (v3), 2-h PG (v2), HbA1c (v2), HOMA-IR (v1), glucose (v1) , HOMA-IR (v3) and triglycerides (v3) respectively.

**Supplementary figure 1a**


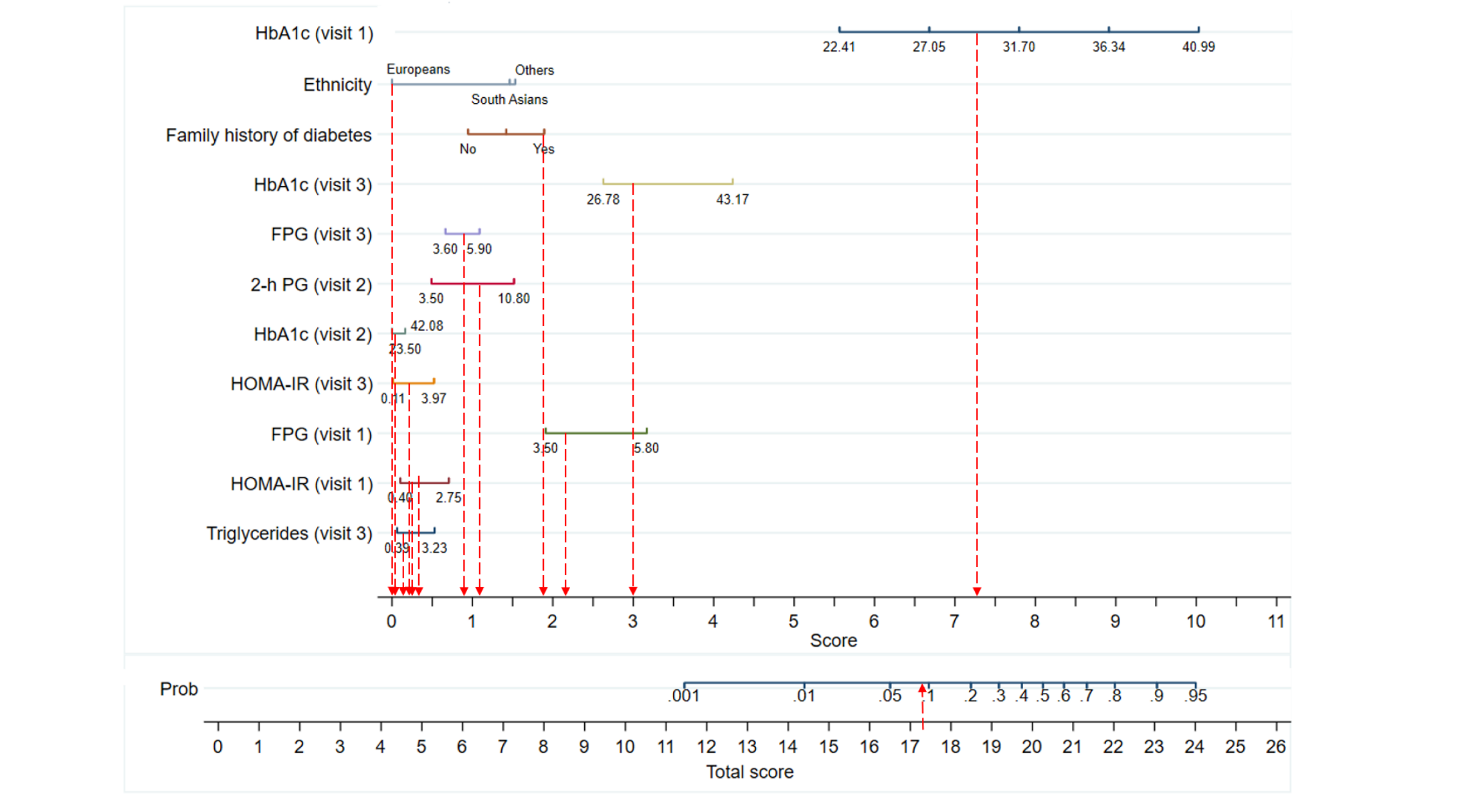


Nomogram based on Case 1: One woman with European origin (Score 0.0). HbA1c 29 mmol/mol (Visit 1) (Score 7.3). Family history of diabetes (yes) (Score 1.9). HbA1c 31 mmol/mol (Visit 3) (Score 3.0). FPG 4.5 mmol/L (Visit 3) (Score 0.9). 2-hour PG 5.6 mmol/L (Visit 2) (Score 1.1). HbA1c 28 mmol/mol (Visit 2) (Score 0.1). HOMA-IR 1.0 (Visit 3) (Score 0.2). FPG 4.0 mmol/L (Visit 1) (Score 2.2). HOMA-IR 0.9 (Visit 1) (Score 0.3). Triglycerides 0.9 mmol/L (Visit 3) (Score 0.3). Total score 17.3. This total score gives a probability of prediabetes or diabetes of approximately 10 %.

**Supplementary figure 1b**


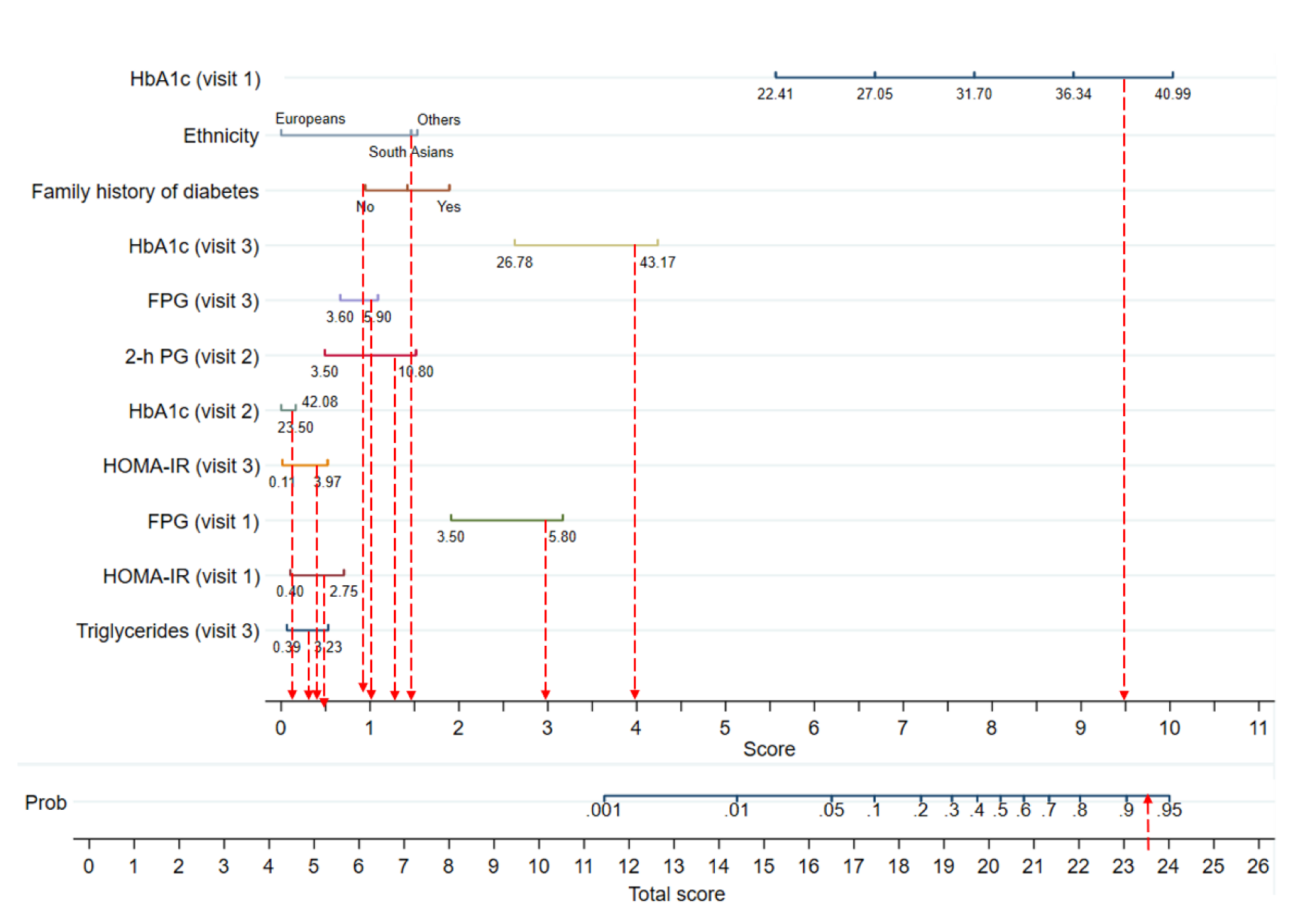


Nomogram based on Case 2: One woman with South Asian origin (Score 1.4). HbA1c 38 mmol/mol (Visit 1) (Score 9.5). Family history of diabetes (no) (Score 1.4). HbA1c 42 mmol/mol (Visit 3) (Score 4.0). FPG 5.6 mmol/L (Visit 3) (Score 1.1). 2-hour PG 8.0 mmol/L (Visit 2) (Score 1.3). HbA1c 41 mmol/mol (Visit 2) (Score 1.1). HOMA-IR 3.5 (Visit 3) (Score 0.4). FPG 5.5 mmol/L (Visit 1) (Score 3.0). HOMA-IR 2.5 (Visit 1) (Score 0.5). Triglycerides 2.7 mmol/L (Visit 3) (Score 0.3). Total score 23.6. This total score gives a probability of prediabetes or diabetes of approximately 92.5 %.
